# Supplementary figures and images for: Effective differentiation of double negative thymocytes requires high fidelity replication of mitochondrial DNA in an age dependent manner
Source: Front Immunol. 2023 Mar 20;14:1128626. doi: 10.3389/fimmu.2023.1128626 (PMC10067910; doi:10.3389/fimmu.2023.1128626)

## PolG Expression

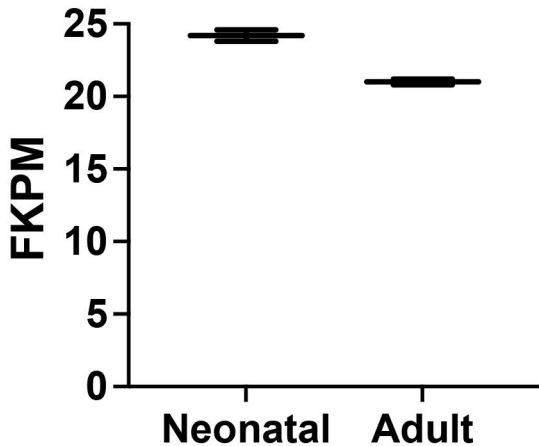

# Lymph node

# Spleen

- PolG*<sup>+/+</sup>
- PolG*<sup>+/D257A</sup>
- PolG*<sup>D257A/D257A</sup>

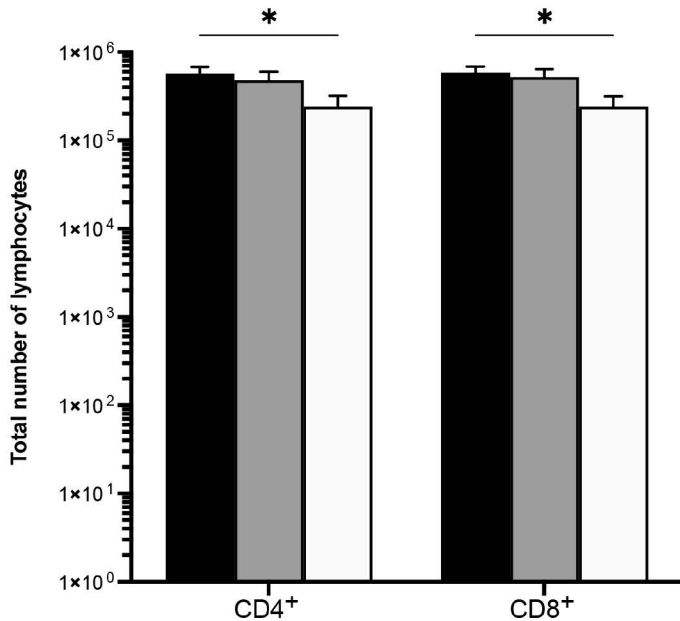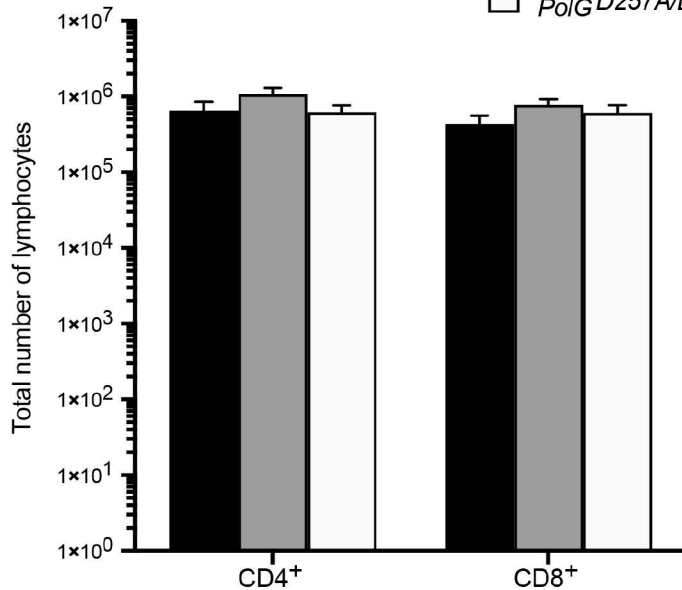

Supplement: Supplementary Figure 1 — No difference in PolG mRNA expression in neonatal an adult thymus. RNA-Sequencing data from neonatal (6-8 days old) and adult gBT-1 thymocytes (2-4 months old, GSE80597) was analyzed for expression of polg. Results not statistically significant. [file DataSheet_1.pdf]
